# Editorial: Inhibition strategies on the formation of Maillard reaction products in food

**DOI:** 10.3389/fnut.2023.1162097

**Published:** 2023-04-04

**Authors:** Qian Wu, Chunxue Yang, Ruojie Zhang

**Affiliations:** ^1^Cooperative Innovation Center of Industrial Fermentation (Ministry of Education & Hubei Province), Key Laboratory of Fermentation Engineering (Ministry of Education), National “111” Center for Cellular Regulation and Molecular Pharmaceutics, Hubei Key Laboratory of Industrial Microbiology, Hubei University of Technology, Wuhan, Hubei, China; ^2^School of Public Health (Shenzhen), Sun Yat-sen University, Shenzhen, China; ^3^Reynolds American (United States), Winston-Salem, NC, United States

**Keywords:** editorial, inhibition strategy, formation, Maillard reaction, food

The Maillard reaction (MR), also known as the non-enzymatic browning reaction, is a complex network of continuous and parallel reactions triggered by the condensation of the amino group of proteins, or amino acids, with the sugar carbonyl group, resulting in the formation of a multitude of compounds known as MR products (MRPs) (1). MRPs of various chemical structures and from different precursors, including undesirable carcinogens such as advanced glycation end-products (AGEs), heterocyclic amines (HAs), acrylamide (AA), and 5-hydroxymethylfurfural (HMF), can be formed as the MR continues (2). In numerous diseases, such as diabetes, neuropathy, atherosclerosis, nephropathy, retinopathy, and chronic renal illness, the accumulation of AGEs has been proposed as a pathogenic mechanism of inflammation, oxidative stress, and structural tissue damage leading to chronic vascular issues.

The focus of this Research Topic was to improve the field's understanding of the MR; additional objectives were to verify the effect of polyphenols on the MR in food, with special emphasis on inhibition of hazardous food substances and future challenges; to provide an extensive overview of the field; and to identify novel research areas to ensure food safety and human health. Five studies were included in this Research Topic, two of which examined the topic from a food processing perspective and one from an *in vivo* perspective. Two others present descriptions of the Maillard reaction in terms of biotransformation as well as collaterals separately.

To determine whether lotus seedpod oligomeric procyanidins (LSOPCs) have an effect on food sensory quality and the MR in foods, Chen et al. added various amounts of LSOPCs to hard biscuits. The findings of the experiments showed that adding LSOPCs can successfully inhibit the MR in hard biscuits while also enhancing their flavor. Cookies were chosen as a food model by Wu et al., who came to the same conclusion.

Feng et al. primarily investigated whether the MR could be inhibited more efficiently by litchi pericarp oligomeric procyanidins (LPOPCs) during lactic acid bacteria (LAB) biotransformation. The findings showed that the antioxidant activity of LPOPCs was significantly increased after incubation with LAB, but the inhibitory effect on AGEs was decreased in both simulated food processing and gastrointestinal digestion systems *in vitro*.

Kang et al. elucidated the pathogenetic roles of AGEs in the progression of diabetic retinopathy (DR), including metabolic abnormalities, lipid peroxidation, structural and functional alterations, and neurodegeneration.

Finally, Wang et al. explored the effects of oligopeptides and reducing sugars on the MR at body temperature. They concluded that histidine but alanine in carnosine may contribute to the glucose lowing effect. The findings of their study also indicated that carnosine shows potential as a bioactive peptide to control blood sugar levels in diabetes without generating adverse effects.

In conclusion, we believe that this compilation of articles will provide valuable information to readers and inspire and motivate young researchers and authors working in the area of food chemistry and the MR.

## Author contributions

QW wrote the introduction and the conclusion. CY wrote the middle section with comments on the cited articles and references. RZ revised the manuscript critically. All authors contributed to the manuscript and approved the submitted version.

## References

[1] Khan M Liu H Wang J Sun B. Inhibitory effect of phenolic compounds and plant extracts on the formation of advance glycation end products: a comprehensive review. Food Res Int. (2020) 130:108933. 10.1016/j.foodres.2019.108933 32156381

[2] Xu Y Li HX Liang J Ma JN Yang J Zhao XJ. High-throughput quantification of eighteen heterocyclic aromatic amines in roasted and pan-fried meat on the basis of high performance liquid chromatography-quadrupole-orbitrap high resolution mass spectrometry. Food Chem. (2021) 361:11. 10.1016/j.foodchem.2021.130147 34051597

